# Inactivation of EGFR/ERK/NF‐κB signalling associates with radiosensitizing effect of 18β‐glycyrrhetinic acid on progression of hepatocellular carcinoma

**DOI:** 10.1111/jcmm.17760

**Published:** 2023-05-12

**Authors:** Yu‐Chang Liu, Cheng Hsun Lin, Kuan‐Tin Chen, De‐Wei Lai, Fei‐Ting Hsu

**Affiliations:** ^1^ Department of Radiation Oncology Chang Bing Show Chwan Memorial Hospital Changhua Taiwan; ^2^ Department of Radiation Oncology Show Chwan Memorial Hospital Changhua Taiwan; ^3^ Department of Medical Imaging and Radiological Sciences Central Taiwan University of Science and Technology Taichung Taiwan; ^4^ Department of Radiation Oncology National Yang Ming Chiao Tung University Hospital Yilan Taiwan; ^5^ Experimental Animal Center, Department of Molecular Biology and Cell Research Chang Bing Show Chwan Memorial Hospital Changhua Taiwan; ^6^ Department of Pharmacy, College of Pharmacy and Health Care Tajen University Pingtung Taiwan; ^7^ Department of Nursing Central Taiwan University of Science and Technology Taichung Taiwan; ^8^ Department of Biological Science and Technology China Medical University Taichung Taiwan

**Keywords:** 18β‐Glycyrrhetinic acid, epidermal growth factor receptor, ERK, hepatocellular carcinoma, NF‐κB, radiotherapy

## Abstract

Hepatocellular carcinoma (HCC) is recognized as the fifth most common cancer and the third most common cause of death in Asian population. Studies reported that HCC is relatively insensitive to radiotherapy (RT); thus, considering how to sensitize HCC to RT is worth to be elucidated. Epidermal growth factor receptor (EGFR)‐mediated signalling transduction plays the important role in regulating treatment efficacy of HCC. An active compound, 18beta‐glycyrrhetinic acid (18β‐GA), has been reported to own anti‐tumour effect. However, whether 18β‐GA possess RT sensitization ability in HCC remains unclear. Here, we used RNA data from TCGA‐LIHC (Liver hepatocellular carcinoma) to identify the role between EGFR/ERK/nuclear factor kappa B (NF‐κB) signalling and RT by radiosensitivity index (RSI) analysis. We suggested that patients with activated NF‐κB signalling may show resistance to RT treatment, whereas combining 18β‐GA may reinforce RT efficacy in a Hep3B‐bearing animal model. 18β‐GA combined with RT showed superior tumour inhibition capacity as compared to monotherapy and even reached similar efficacy as erlotinib combined with RT. Treatment promotion of RT by 18β‐GA in HCC is not only through diminishing RT‐induced EGFR/ERK/NF‐κB signalling but also promoting RT‐induced apoptosis pathways. 18β‐GA may act as radiosensitizer through inactivating EGFR‐mediated HCC progression and inducing caspase‐dependent apoptosis signalling.

## 1 INTRODUCTION

Radiotherapy (RT) techniques such as intensity‐modulated RT (IMRT), image‐guided RT (IGRT), hypo‐fractionated RT (HFRT), and stereotactic body RT (SBRT) can improve the outcome of patients with unresectable hepatocellular carcinoma (HCC) due to accurate delivery of high radiation dose to tumour while avoiding non‐tumorous hepatic tissue irradiation.^1^, ^2^ In addition to the advancement of RT technology, strategy to combine RT with other therapeutic modalities is crucial for further treatment result improvement of HCC.^3^

A number of oncogenic kinases and transcription factors such as epidermal growth factor receptor (EGFR), extracellular signal‐regulated kinase (ERK), activator protein 1, signal transducer and activator of transcription 3 (STAT3), and nuclear factor‐kappaB (NF‐κB) alleviate growth inhibition of RT by upregulating radioresistance in cancer cells.^4^, ^5^, ^6^, ^7^ The combination of sorafenib (a receptor tyrosine kinase inhibitor) and RT has been shown as an effective strategy to significantly prolong survival of patients with HCC.^8^ Suppression of ERK, NF‐κB and STAT3 activity may be involved in the anti‐HCC effect of sorafenib combined with RT.^9^, ^10^

Chinese herbal medicine (CHM) has been recognized as a complementary therapy and can be used for treatment of cancers. Patients with HCC have an increased survival rate from addition of CHM to standard therapy.^11^, ^12^ Demonstrated by cell and animal models, natural compounds extracted from medical plants evoke tumour regression through suppressing of oncogenic pathways, interference of DNA repair mechanism, induction of apoptosis and cell cycle arrest.^13^, ^14^, ^15^ In addition, antitumor properties of natural compounds may also contribute to the enhancement of RT efficacy in HCC cells.^16^

Several natural compounds exhibit both radioprotective and radiosensitive properties not only mitigating radiation‐induced injury in normal tissues but also enhancing anti‐cancer effects of radiation.^17^ For instance, curcumin, a flavonoid isolated from *Curcuma longa*, has been indicated to boost radiation‐inhibited growth of HCC through NF‐κB inactivation as well as attenuating radiation‐induced pneumonitis by downregulation of dual oxidase 1 and 2 expression.^18^, ^19^ 18β‐Glycyrrhetinic acid (18β‐GA), an active compound obtained from medical herb licorice, possesses antioxidative, anti‐inflammatory and antimicrobial capacities.^20^ 18β‐GA was also found to suppress growth and invasion of HCC by inducing apoptosis and targeting STAT3.^21^, ^22^ In addition, 18β‐GA has been indicated to restraint radiation‐induced skin injury via suppression of mitogen‐activated protein kinase (MAPK) and NF‐κB signalling.^23^ However, whether 18β‐GA sensitizes HCC to radiation is ambiguous. The main goal of present study was to evaluate anti‐HCC efficacy and mechanism of 18β‐GA in combination with RT.

## 2 MATERIALS AND METHODS

### 2.1 Reagents

The following reagents were all purchased from Sigma‐Aldrich, including 18β‐GA, erlotinib, DMSO and (3‐[4,5‐dimethylthiazol‐2‐yl]‐2,5‐diphenyltetrazolium bromide) MTT powder.

### 2.2 Cell culture and cell viability assay

Human Hep3B and Huh7 cells utilized in this study were purchased from Bioresource Collection and Research Center (BCRC, Hsinchu, Taiwan) and given by Dr Chiao‐Fang Teng from China Medical University (Taichung, Taiwan). Cells was maintained in Dulbecco's modified Eagle's medium‐high glucose (HyClone), containing 10% serum, 1% penicillin/streptomycin solution at 37°C and 5% CO_2_ incubator. Hep3B and Huh7 cell viability was assayed by MTT after treating with 0–300 μM of 18β‐GA for 24 and 48 h.^24^

### 2.3 Radiosensitivity index (RSI) analysis

In total, 426 patients' RNA sequence data from TCGA‐LIHC were used for RSI analysis. For RSI analysis, the patients' expression level of EGFR, ERK, NF‐κB, respectively, in the first quartile (the lowest 25% of numbers, *n* = 106) was defined as the low expression and the fourth quartile (the highest 25% of numbers, *n* = 106) was defined as high expression levels. The RSI was calculated according to the following formula^25^:

RSI = ‐ 0.0098009 × AR + 0.0128283 × cJun+0.0254552 × STAT1 – 0.0017589 × PKC – 0.0038171 × RelA + 0.1070213 × cABL – 0.0002509 × SUMO1–0.0092431 × PAK2 – 0.0204469 × HDAC1 – 0.0441683 × IRF1.

### 2.4 Western blotting assay

In brief, proteins were collected from Hep3B and Huh7 cells after 100 and 200 μM of 18β‐GA treatment for 48 h. Proteins were separated by 8%–12% SDS‐page and transferred onto PVDF membrane for probing primary antibody. Relative secondary antibodies were finally conjugated and followed with Immobilon Western Chemiluminescent HRP Substrate for visualizing.^26^ Primary antibodies are listed as follows: EGFR (Tyr1068) (Cell Signaling), EGFR (Elabscience), Phospho‐p44/42 MAPK (Erk1/2) (Cell Signaling), ERK (Santa Cruz Biotechnology), NF‐κB (Ser536) (Cell Signaling Technology), NF‐κB (Elabscience) and β‐actin (Santa Cruz Biotechnology).

### 2.5 Nuclear translocation analysis of NF‐κB

Hep3B and Huh7 cells were seeded on coverslips overnight and followed with different concentrations of 18β‐GA treatment for 48 h. After treatment, coverslips were fixed by 3.7% paraformaldehyde, permeabilized by 0.1% Triton‐X100, blocked by 1% bovine serum albumin, stained by NF‐κB primary antibody and conjugated with Fluor®488 conjugated‐secondary goat‐anti‐rabbit antibody. Fluorescence images was acquired by Revolve Fluorescence Microscope by Echo and quantified by ImageJ software version 1.50 (National Institutes of Health).

### 2.6 Hep3B‐bearing animal model

Animal experiment was approved by Institutional Animal Care and Use Committee (IACUC) from China Medical University with approval ID: CMUIACUC‐2020‐373. CAnN.Cg‐*Foxn1* ^*nu*^/CrlNarl (nude mice) was purchased from National Laboratory Animal Center, Taipei, Taiwan. Five million Hep3B with 30% matrigel was inoculated into mice right flank for establishing Hep3B animal model. Mice were divided into five groups after the average tumour size reached 100 mm^3^, including (1) control group (0.1% DMSO in 100 μL PBS daily gavage), (2) 18β‐GA‐treated group (10 mg/kg in 100 μL PBS daily gavage), (3) radiation therapy group (RT, 6 Gy local irradiation with single administration), (4) 18β‐GA plus RT, and (5) erlotinib (10 mg/kg in 100 μL PBS daily gavage) plus RT. Tumour size was recorded every 3 days and calculated by formula from Tomayko and Reynolds (volume = length × width^2^ × 0.523).^27^

### 2.7 Haematoxylin and eosin (H&E) staining on normal tissue for pathology evaluation

Mice spleen, heart, intestine, liver and kidney were collected and sliced for H&E staining.^28^ The staining procedure was performed by Bio‐Check Laboratories Ltd. (New Taipei City, Taiwan). Tissue images were acquired by TissueFAXS platform (TissueGnostics) at 200 × magnification.

### 2.8 Immunohistochemistry (IHC) staining on tumour tissue for protein evaluation

Tumour section acquired from mice was collected for IHC staining with IHC Select^®^ HRP/DAB reagents (EMD Millipore, Merck KGaA). After staining, tumour‐stained sections were imaged at 200 × magnification by TissueFAXS platform (TissueGnostics) and quantified by StrataQuest software using BF Area total 2 markers tool.^29^

### 2.9 Statistical analysis

Data were analysed by the software GraphPad Prism 7, and presented as mean ± standard deviation. Statistical calculation was performed by Student's *t*‐test or one‐way analysis of variance (anova). The difference between control and treatment group was recognized as statistically significant when *p* < 0.05.

## 3 RESULTS

### 3.1 18β‐GA may sensitize HCC to RT via inactivating EGFR/ERK/NF‐κB signalling

**FIGURE 1:**
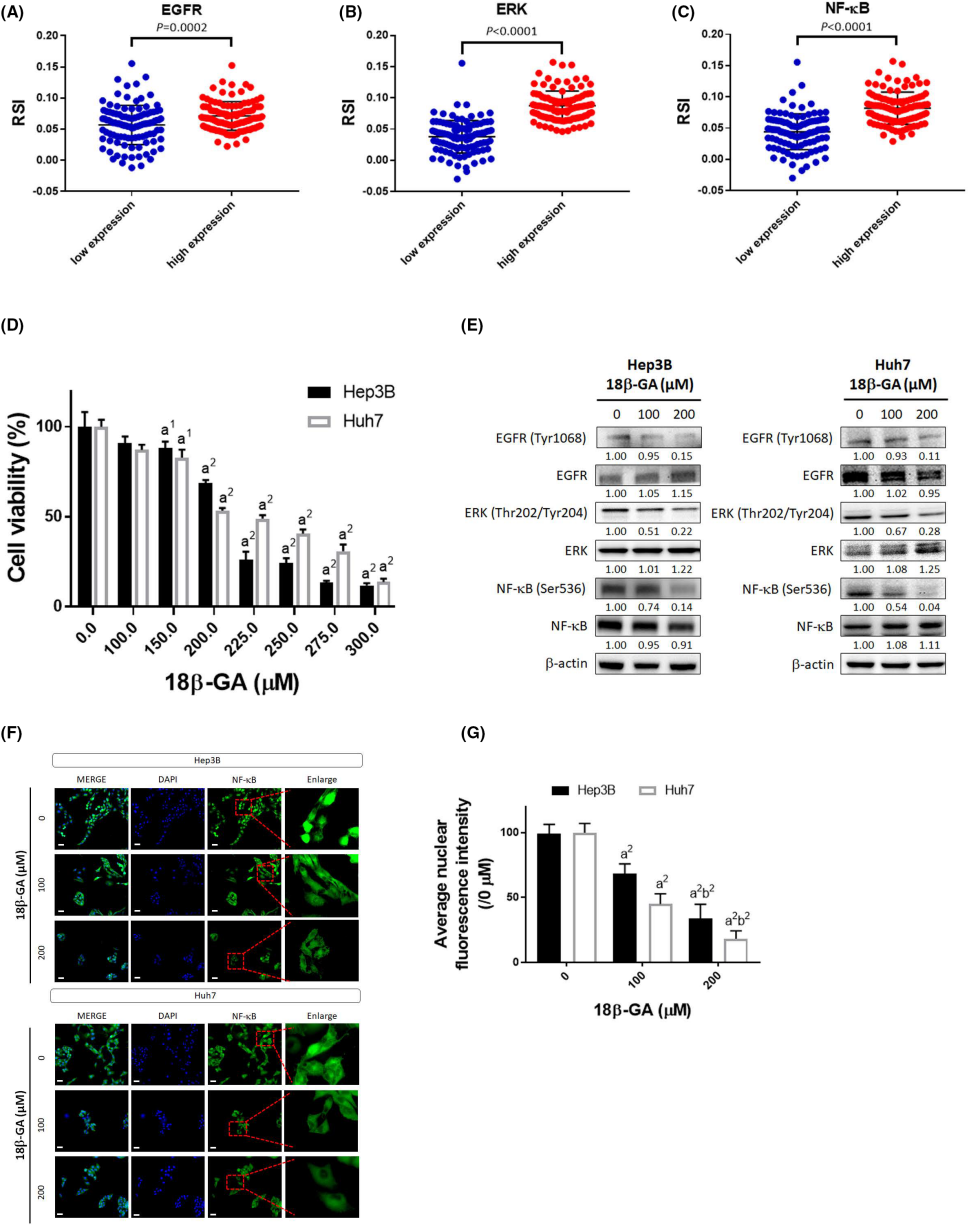
Cytotoxicity efficacy of RT was enhanced by 18β‐GA via targeting EGFR/ERK/NF‐κB signalling. The RSI analysis based on different expression levels of (A) EGFR, (B) ERK and (C) NF‐κB in HCC patient samples are displayed. (D) The cytotoxicity effect of 48 h treatment of 0–300 μM 18β‐GA on Hep3B and Huh7 cells was assayed by MTT. (E) The expression level of EGFR, ERK and NF‐κB after 0, 100 and 200 μM 18β‐GA treatment is assayed by Western blotting. (F, G) The NF‐κB nuclear translocation after 0, 100 and 200 μM 18β‐GA treatment is assayed by IF staining. (a^1^ *p* < 0.05, a^2^ *p* < 0.01 vs. 0 μM 18β‐GA; b^2^ *p* < 0.01 vs. 100 μM 18β‐GA in both Hep3B and Huh7).

A total of 424 patients' RNA sequence data were used to perform EGFR, ERK and NF‐κB expression level analysis, respectively. These patients were divided into two groups based on EGFR, ERK and NF‐κB expression level, top 25% expression level (defined as high expression level) and bottom 25% expression level (defined as low expression level) was sampled for RSI analysis. As indicated in Figure 1A–C, the RSI value was decreased in patients with lower expression level of EGFR (*p* = 0.0002), ERK (*p* < 0.0001), NF‐κB (*p* < 0.0001). These results suggested that patients with lower expression level of EGFR, ERK, NF‐κB may relatively be sensitized to RT. Here, we indicated that 18β‐GA may not only induce cytotoxicity (Figure 1D) but also suppress the phosphorylation of EGFR, ERK, NF‐κB of both Hep3B and Huh7 cells (Figure 1E). Additionally, 18β‐GA may also suppress the nuclear translocation of NF‐κB in a dose‐dependent manner (Figure 1F,G). This could imply that 18β‐GA may exhibit RT sensitization potential via inactivating EGFR, ERK and NF‐κB signalling.

### 3.2 18β‐GA may enhance anti‐HCC efficacy of RT in HCC‐bearing animal model

**FIGURE 2:**
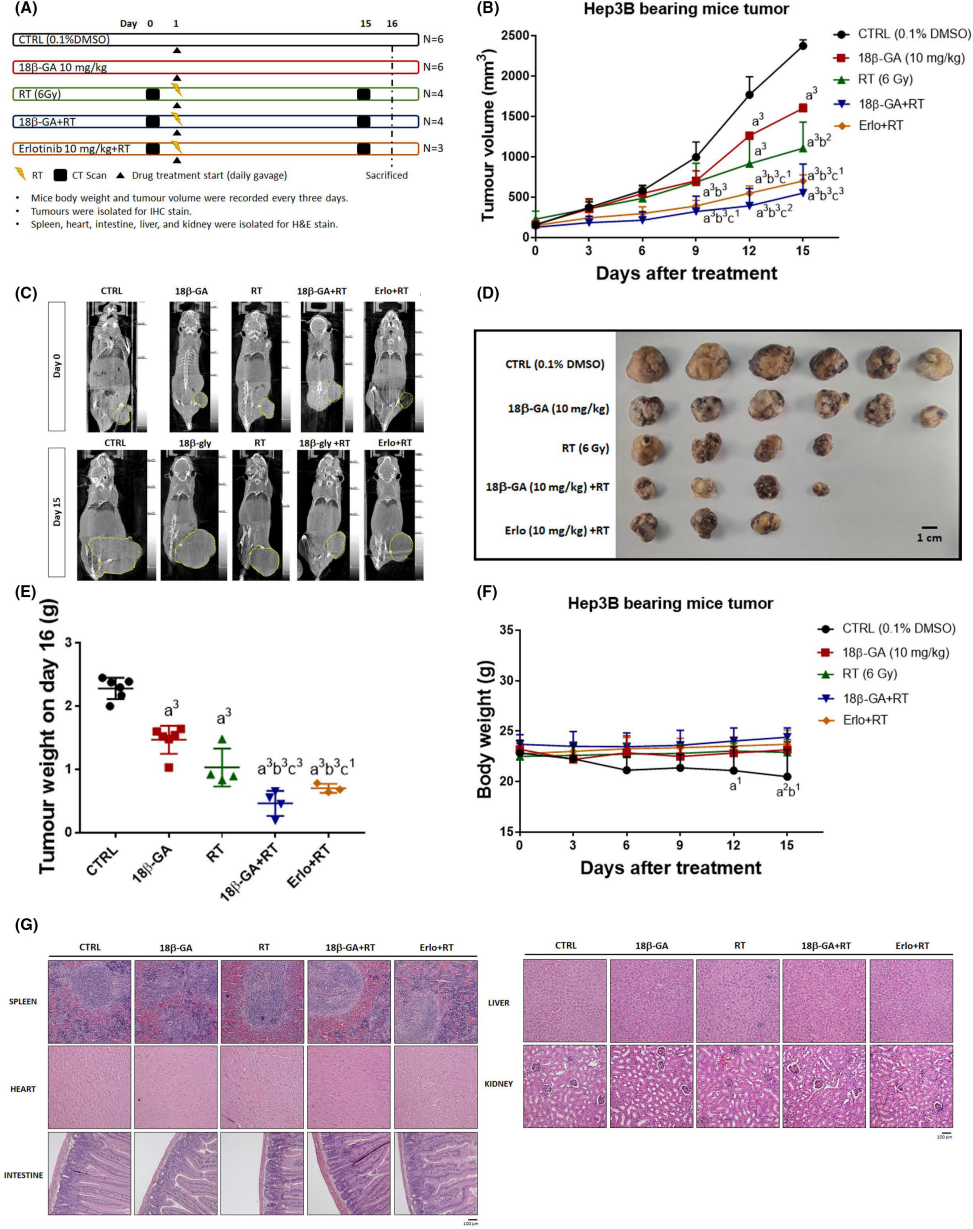
HCC tumour inhibition efficacy of RT was enhanced by 18β‐GA in vivo. (A) The animal experimental flow chart is displayed. (B) Tumour growth pattern, (C) CT images, (D) extracted tumour and (E) extracted tumour weight of different treatment are shown. (F) Body weight and (G) H&E staining results are displayed (a^3^ *p* < 0.0001 vs. Control; b^2^ *p* < 0.01, b^3^ *p* < 0.0001 vs. 10 mg/kg 18β‐GA; c^1^ *p* < 0.05, c^2^ *p* < 0.01, c^3^ *p* < 0.0001 vs. 6 Gy RT) (Erlo, erlotinib; RT, radiotherapy; 18β‐GA, 18β‐glycyrrhetinic acid).

To elucidate whether 18β‐GA may accurately sensitize HCC to RT treatment, we established Hep3B animal model. Mice was divided into five treatments group, wherein erlotinib (EGFR inhibitor, erlo)‐combined RT was regarded as the positive control group (Figure 2A). Tumour progression effect was effectively suppressed by 18β‐GA combined with RT and erlotinib‐combined RT group as compared to monotherapy (Figure 2B). The mean tumour growth time (MTGT) and mean tumour growth delay time (MTGDT) was higher in 18β‐GA + RT than Erlo + RT (Table 1). Additionally, the enhancement treatment efficacy ratio of 18β‐GA + RT as compared to18β‐GA alone or RT alone is also indicated as 3.41 and 2.08 times, respectively. The synergistic combination index (CI) of 18β‐GA combined with RT was calculated, presented in Table 2 and showed to be 0.57. The CI smaller than 1 indicated that 18β‐GA combined with RT showed superior effect than monotherapy of 18β‐GA or RT. Furthermore, CT scanning images, extracted tumour images and extracted tumour weight from each group all indicated the superior tumour inhibition effect of 18β‐GA combined with RT as compared to monotherapy (Figure 2C–E). Due to the progression of tumour, the body weight of the untreated group showed a marked reduction as compared to the treated group (Figure 2F). Normal tissue pathology, including spleen, heart, intestine, liver and kidney, does not present obvious difference between the treated and untreated groups (Figure 2G). Both body weight and pathology results supported that a combination of 18β‐GA with RT may not generate general toxicity in HCC‐bearing mice. Taken together, 18β‐GA may significantly boost anti‐HCC efficacy of RT.

**TABLE 1:** Mean tumour growth time, delay time, inhibition rate, and enhancement ratio in Hep3B tumour‐bearing mice after treatment with 18β‐GA, RT, the combination of 18β‐GA and RT, and the combination of erlotinib and RT.

	Hep3B				
	Control	18β‐GA	RT	18β‐GA + RT	Erlo+RT
MTGT (day)^a^	6.76	10.42	17.08	35.48	27.23
MTGDT(day)^b^	NA	3.66	10.33	28.72	20.47
ER^C^	NA	3.41	2.08	–	NA

NA, not available.

^a^ Mean tumour growth time (MTGT): the time at which the tumour volume reached 1100 mm^3^.

^b^ Mean tumour growth delay time (MTGDT): the mean tumour growth time of the treated group minus that of the control group.

^C^ Enhancement ratio (ER): the mean growth inhibition rate of the 18β‐GA and RT combination group/the mean growth inhibition rate of the 18β‐GA or RT group.

**TABLE 2:** Mean tumour growth inhibition rate and combination index in Hep3B tumour‐bearing mice after treatment with 18β‐GA, RT and the combination of both.

	18β‐GA		RT		Combination		
Xenografts	MGIR^a^		MGIR^a^		Expected^b^	Observed^c^	Index^d^
Hep3B	0.33		0.53		0.59	0.77	0.57

^a^ Mean growth inhibitory rate (MGIR): 1‐(the 15th day's mean tumour volume ratio of treated group/the 15th day's mean tumour volume ratio of the control group).

^b^ Expected growth inhibitory rate: inhibition rates of combination minus the multiplication of both 18β‐GA and inhibition rates.

^c^ Observed growth inhibitory rate: growth inhibition rate of 18β‐GA combined with RT (combination group).

^d^ Index value was calculated by (1‐ MGIR of combination)/(1‐Expected growth inhibitory rate). An index <1.0 indicates a synergistic effect.

### 3.3 18β‐GA effectively revoked RT‐induced EGFR/ERK/NF‐κB signalling transduction and its related metastasis protein expression

**FIGURE 3:**
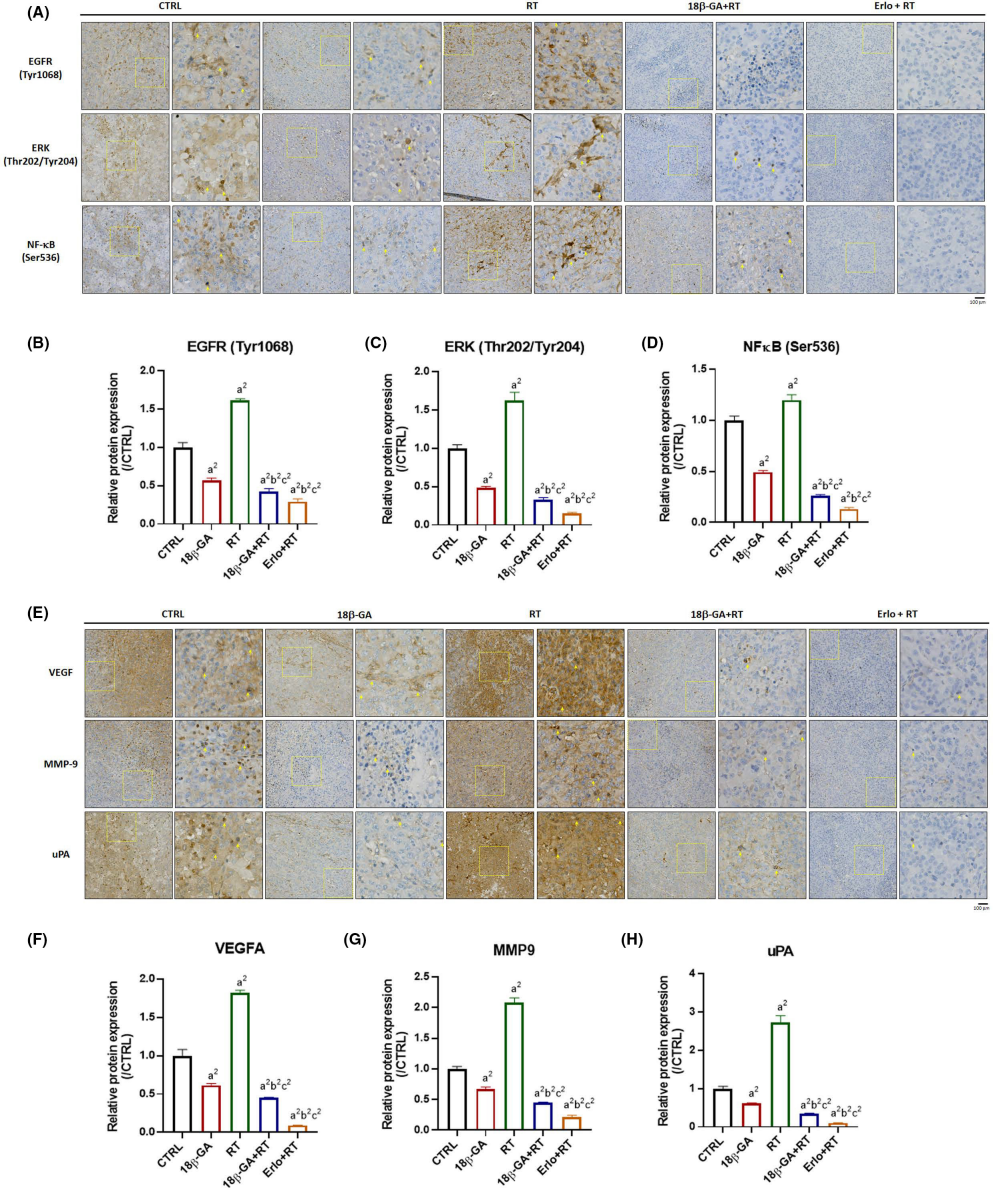
RT induced EGFR/ERK/NF‐κB signalling and its mediated metastasis‐associated proteins were diminished by 18β‐GA. (A, E) IHC expression pattern and quantification results of (B) EGFR Tyr 1068, (C) ERK Thr202/Tyr204, (D) NF‐κB Ser536, (F) VEGFA, (G) MMP‐9 and (H) uPA are presented. Each yellow square highlights the enlarged area of the IHC staining image, which has been magnified by a factor of nine. (a^2^ *p* < 0.01 vs. Control; b^2^ *p* < 0.01 vs. 10 mg/kg 18β‐GA; c^2^ *p* < 0.01 vs. 6 Gy RT) (Erlo, erlotinib; RT, radiotherapy; 18β‐GA: 18β‐glycyrrhetinic acid).

To confirm whether 18β‐GA enhanced anti‐HCC efficacy of RT is associated with the inactivation of EGFR/ERK/NF‐κB signalling, we performed IHC staining on tumour samples. Figure 3A demonstrates that the expression patterns of phospho‐EGFR (EGFR Tyr 1068), phospho‐ERK (ERK Thr202/Tyr204) and phospho‐NF‐κB (NF‐κB Ser536) were all induced by RT treatment; however, they were suppressed by combining with 18β‐GA. A markedly suppressed effect on the EGFR/ERK/NF‐κB pathway was found in mice tumour tissue treated with both 18β‐GA and RT as compared to monotherapy (Figure 3B–D). In addition, EGFR/ERK/NF‐κB‐mediated metastasis‐related proteins, such as VEGFA, MMP‐9 and uPA, were also found to be decreased by 18β‐GA (Figure 3E–G). As illustrated in Figure 3, not only RT‐induced EGFR/ERK/NF‐κB signalling, but the metastasis‐related proteins mediated by these pathways were effectively revoked by 18β‐GA treatment.

### 3.4 18β‐GA effectively abrogated RT‐induced anti‐apoptosis protein expression and boosted RT‐induced apoptosis protein expression

**FIGURE 4:**
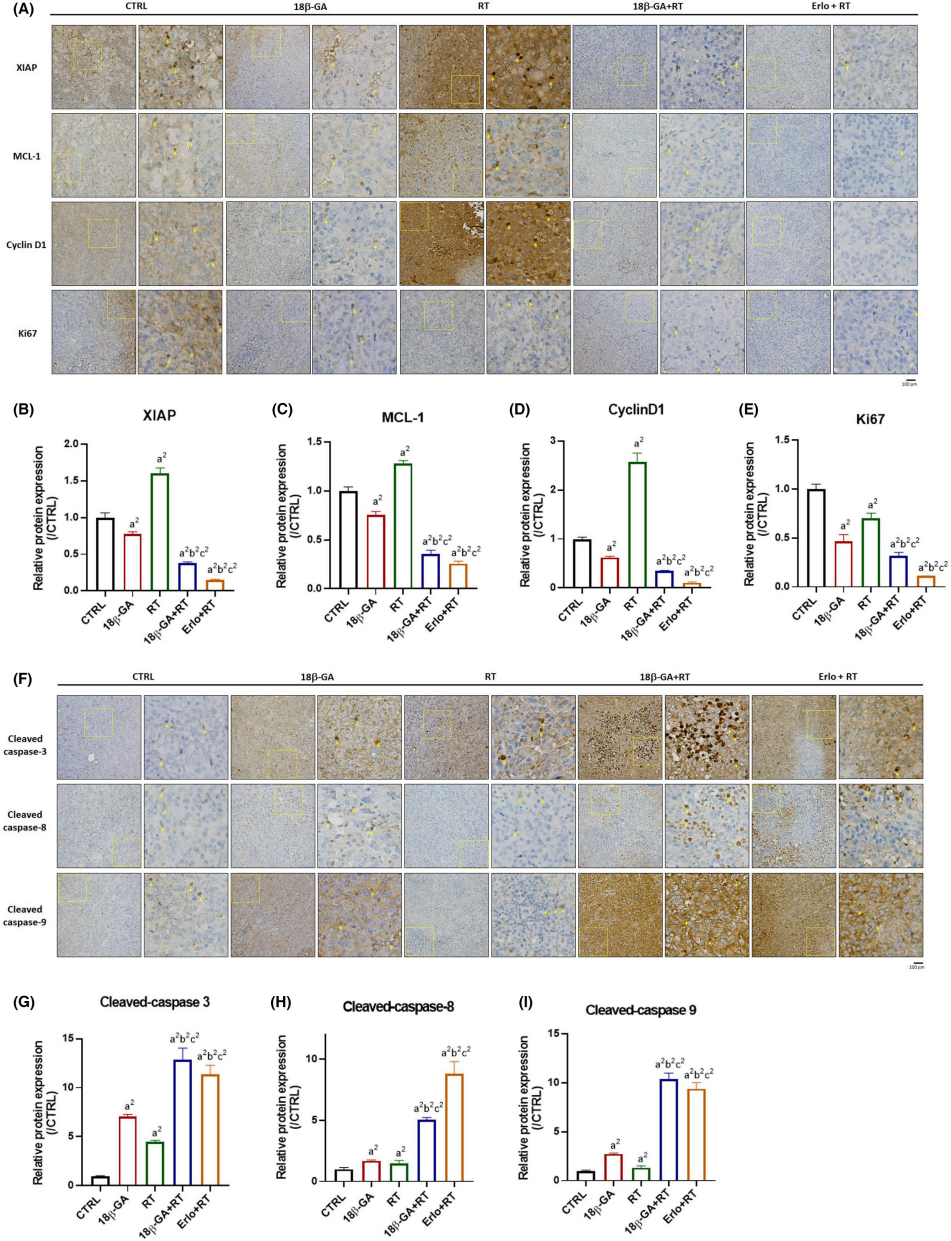
RT induced apoptosis protein expression was strengthening by 18β‐GA. (A, F) IHC expression pattern and quantification results of (B) XIAP, (C) MCL‐1, (D) Cyclin‐D1, (E) Ki‐67, (G) cleaved caspase‐3, (H) cleaved caspase‐8 and (I) cleaved caspase‐9 are presented. Each yellow square highlights the enlarged area of the IHC staining image, which has been magnified by a factor of nine (a^2^ *p* < 0.01 vs. Control; b^2^ *p* < 0.01 vs. 10 mg/kg 18β‐GA; c^2^ *p* < 0.01 vs. 6 Gy RT) (Erlo, erlotinib; RT, radiotherapy; 18β‐GA: 18β‐glycyrrhetinic acid).

Next, we identified whether anti‐apoptosis‐related proteins and apoptosis‐related proteins were affected by 18β‐GA combined with RT. As indicated in Figure 4A, the anti‐apoptosis proteins XIAP, MCL‐1, Cyclin‐D1 that induced by RT were abrogated by combining with 18β‐GA. The expression levels of XIAP, MCL‐1 and Cyclin‐D1 were markedly decreased in 18β‐GA combined with the RT sample (Figure 4B–D). Ki‐67, which represented as tumour proliferation, was found to be significantly increased in the combination group as compared to monotherapy (Figure 4E). Moreover, RT‐induced apoptosis‐related protein including cleaved caspase‐3, ‐8 and ‐9 were all promoted by 18β‐GA as well (Figure 4F–I). In sum, 18β‐GA may revoke RT‐induced XIAP, MCL1 and Cyclin‐D1 expression but reinforce RT‐induced Ki‐67 and apoptosis‐related markers.

## 4 DISCUSSION

The Epidermal growth factor receptor (EGFR) is a receptor tyrosine kinase that, upon activation, upregulates downstream signalling cascades that initiate oncogenic processes, such as cancer cell proliferation, anti‐apoptosis, angiogenesis and invasion.^30^ Abundant EGFR expression has been shown to render mouse ovary carcinoma cell insensitivity to radiation.^4^ Overexpression of EGFR is found in HCC and is correlated with poor patient outcome.^31^ EGFR signalling has been shown to elicit the resistance of HCC to tyrosine kinase inhibitors but the relationship between EGFR expression and radioresistance of HCC remains ambiguous.^32^, ^33^ The information obtained from the TCGA databank indicates that HCC patients with high expression of EGFR responded had a worse response to RT compared to those with low expression (Figure 1A).

The combination of erlotinib (an EGFR inhibitor) with bevacizumab is an effective therapeutic approach for HCC patients who progressed on sorafenib treatment.^34^ Here, we also found that erlotinib as a radiosensitizer significantly enhanced radiation‐inhibited tumour growth in HCC in vivo (Figure 2B–D). EGFR mediates resistance of tumour to radiation through activating downstream oncogenic pathways involved in tumour progression.^35^ ERK and NF‐κB can be activated by EGFR signalling to induce the expression of anti‐apoptotic, proliferation and invasion‐associated proteins.^36^, ^37^ The information retrieved from the TCGA databank presented that high levels of both ERK and NF‐κB reduced survival benefit of RT in HCC patients (Figure 1B,C). Radiation was indicated to trigger the autophosphorylation of EGFR.^38^ We found erlotinib effectively reduced radiation‐induced EGFR, ERK, and NF‐κB phosphorylation (Figure 3A–D).

**FIGURE 5:**
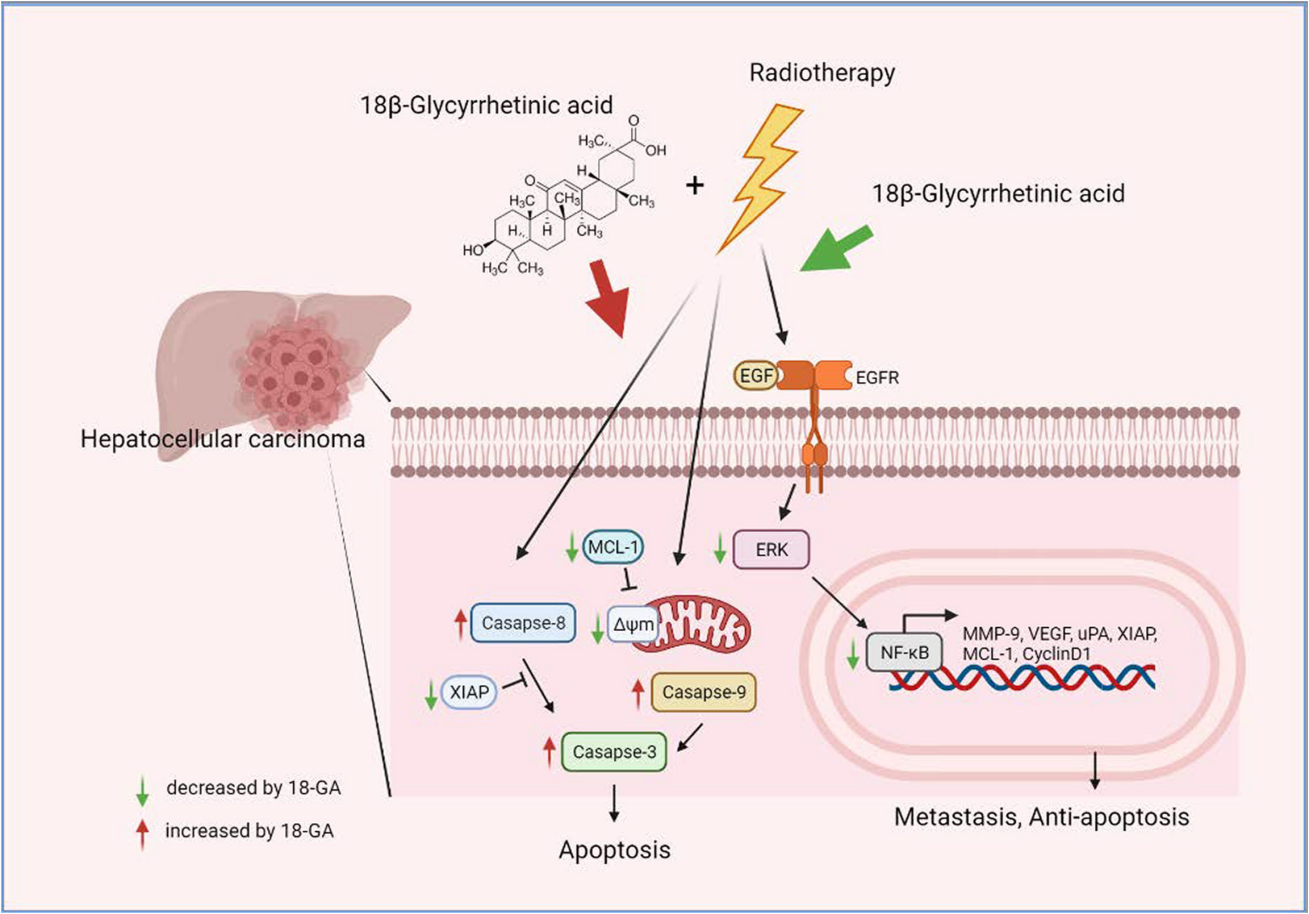
The proposed mechanism of 18β‐GA combined with RT on HCC. Green arrow in figure represented as inhibition effect; on the contrary, red arrow was recognized as induction effect.

18β‐GA was reported to evoke anti‐HCC effect by targeting EGFR.^39^ Our data showed that 18β‐GA inhibited phosphorylation of EGFR, ERK and NF‐κB while effectively attenuated NF‐κB nuclear translocation in Hep3B and Huh7 cells. We verified that 18β‐GA retained anti‐EGFR, ERK and NF‐κB activity in HCC cells (Figure 1E–G). However, whether 18β‐GA upregulated the radiosensitivity of HCC needs to be elucidated. We found that 18β‐GA also significantly potentiated radiation‐inhibited growth of HCC in vivo. Both 18β‐GA and erlotinib effectively enhanced radiation‐induced apoptosis through extrinsic and intrinsic pathways (Figure 4F–I). Notably, 18β‐GA showed the same effect as erlotinib to suppress radiation‐induced EGFR/ERK/NF‐κB signalling transduction and expression of downstream effector proteins (VEGF, MMP‐9, uPA, XIAP, MCL‐1 and Cyclin‐D1) (Figure 5). Expression of the above‐mentioned invasion, anti‐apoptotic and proliferation‐associated proteins is linked to ERK/NF‐κB signalling and involved in radioresistance of tumour cells.^2^, ^40^, ^41^, ^42^, ^43^, ^44^

## 5 CONCLUSION

In conclusion, 18β‐GA as a radiosensitizer promotes radiation effectiveness on inhibition of tumour growth and induction of apoptosis in HCC in vivo. We suggested that blockade of EGFR/ERK/NF‐κB signalling is associated with 18β‐GA‐sensitized HCC to radiation.

## AUTHOR CONTRIBUTIONS

**Yu‐Chang Liu:** Data curation (equal); formal analysis (equal); writing – original draft (equal). **Cheng Hsun Lin:** Data curation (equal); validation (equal); writing – original draft (equal). **Kuan‐Tin Chen:** Conceptualization (equal); data curation (equal); writing – original draft (equal). **De‐Wei Lai:** Data curation (equal); software (equal); validation (equal); writing – original draft (equal). **Fei‐Ting Hsu:** Conceptualization (lead); data curation (equal); funding acquisition (equal); investigation (equal); writing – original draft (equal); writing – review and editing (equal).

## CONFLICT OF INTEREST STATEMENT

No potential competing interest was reported by the authors.

## Data Availability

The data that support the findings of this study are available on request from the corresponding author.
